# LcrG secretion is not required for blocking of Yops secretion in *Yersinia pestis*

**DOI:** 10.1186/1471-2180-8-29

**Published:** 2008-02-08

**Authors:** Laura D Reina, Deanna M O'Bryant, Jyl S Matson, Matthew L Nilles

**Affiliations:** 1 Department of Microbiology and Immunology, University of North Dakota, School of Medicine and Health Sciences, Grand Forks, ND 58202, USA; 2 Department of Microbiology and Immunology, University of Michigan Medical School, Ann Arbor, MI 48109-0620, USA

## Abstract

**Background:**

LcrG, a negative regulator of the *Yersinia *type III secretion apparatus has been shown to be primarily a cytoplasmic protein, but is secreted at least in *Y. pestis*. LcrG secretion has not been functionally analyzed and the relevance of LcrG secretion on LcrG function is unknown.

**Results:**

An LcrG-GAL4AD chimera, originally constructed for two-hybrid analyses to analyze LcrG protein interactions, appeared to be not secreted but the LcrG-GAL4AD chimera retained the ability to regulate Yops secretion. This result led to further investigation to determine the significance of LcrG secretion on LcrG function. Additional analyses including deletion and substitution mutations of amino acids 2–6 in the N-terminus of LcrG were constructed to analyze LcrG secretion and LcrG's ability to control secretion. Some changes to the N-terminus of LcrG were found to not affect LcrG's secretion or LcrG's secretion-controlling activity. However, substitution of poly-isoleucine in the N-terminus of LcrG did eliminate LcrG secretion but did not affect LcrG's secretion controlling activity.

**Conclusion:**

These results indicate that secretion of LcrG, while observable and T3SS mediated, is not relevant for LcrG's ability to control secretion.

## Background

*Yersinia pestis *contains a 75-kilobase (Kb) virulence plasmid called pCD1 that encodes the low calcium response (Lcr) regulon [1]. LcrG is a negative regulator of the *Yersinia *type three-secretion system (T3SS) that is thought to control secretion of the T3SS-secreted effectors [2], collectively termed Yops [1]. The *Yersinia *T3SS is activated by environmental signals [3]; in the presence of calcium, LcrG blocks secretion from the cytoplasm [4], and in the absence of calcium, LcrG is primarily located in the cytosol, with smaller amounts found in association with membranes and secreted into the culture supernatant [4]. LcrG binds another *Yersinia *regulatory protein, LcrV, to unblock secretion via LcrG-LcrV interaction [4]. According to the LcrG-titration model, in the presence of secretion-inducing conditions, LcrQ is exported, causing levels of LcrV to increase relative to the levels of LcrG. The excess LcrV titrates LcrG and relieves LcrG's secretion-blocking activity, possibly by removing LcrG from the secretion complex, which would allow full induction of the LCR and subsequent secretion of Yops [5,6]. Based on the LcrG-titration model, LcrG secretion would not be necessary for LcrG function, accordingly this study addresses the significance of LcrG secretion to LcrG function in *Y. pestis*.

Signals that target Yops to the T3SS apparatus have been localized to the N-terminus of the Yops and to the mRNA [7-11]. However, neither the N-terminus of T3S-substrates nor the mRNA shares a consensus sequence, and the manner in which the T3SS can recognize diverse substrates is unclear [12-14]. Systematic mutagenesis of the presumed secretion signal in the N-terminus of YopE yielded mutants defective in Yop translocation but point mutants that abolished secretion were not identified [10]. Frameshift mutations that allowed the peptide sequences of these signals to remain intact also failed to prevent secretion. Suggesting that the signal that leads to secretion of Yops appears to be encoded in their mRNA rather than the peptide sequence [15]. In the case of YopQ, frameshift mutations were tolerated only when at least 13 codons of the T3SS signal sequence are present [15]. Mutations in the second codon of the secretion signal may abolish synthesis of YopQ, and mutations in the tenth codon may abolish secretion without affecting YopQ's synthesis [15].

In this study, we show that chimeric LcrG proteins with the GAL4 activation domain (from the GAL4 protein of *Saccharomyces cerevisae *[16]) fused to the N-terminus and the C-terminus of LcrG were not secreted intact. These non-secreted LcrG-GAL4AD chimeras appeared to transcomplement a Δ*lcrG3 *strain of *Y. pesti*s, demonstrating retention of LcrG function. This result was extended and confirmed by constructing deletion and substitution mutations affecting amino acids 2–6 in the N-terminus of LcrG. None of the LcrG mutants were affected for LcrG function. However, secretion of some of the functional mutant LcrGs could not be detected, suggesting that secretion of LcrG is not relevant for known LcrG functions.

## Results and Discussion

**Figure 1:**
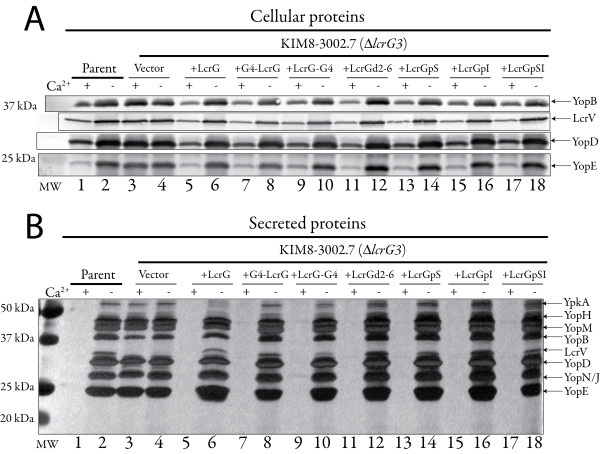
**Transcomplementation of *Y. pestis *Δ*lcrG3 *with LcrG chimeras and N-terminal LcrG mutants**. (A) Immunoblot detection of cellular LcrV, YopB, YopD, and YopE. Cells of *Y. pestis*, wild type (WT) containing plasmid pBAD18 (vector; lanes 1and 2), *Y. pestis *ΔLcrG3 containing plasmids pBAD18 (vector; lanes 3 and 4), pAraG18 (LcrG, lanes 5 and 6), pJM174 (GAL4AD-LcrG; lanes 7 and 8), pLR1 (LcrG-GAL4AD, lanes 9 and 10), pLR2 (LcrGd2-6, lanes 11 and 12), pLR3 (LcrGpS; lanes 13 and 14), pLR4 (LcrGpI, lanes 15 and 16), pLR5 (LcrGpSI, lanes 17 and 18) were separated by SDS-PAGE in a 10.5–14% gradient, 4–20% gradient or 12.5% polyacrylamide gels and immunoblotted. Immunoblots were probed with α-YopB (12.5% gel), α-YopD, α-YopE (10.5–14% gel) and α-LcrV (4–20% gel) primary antibodies. Proteins were visualized with alkaline-phosphatase conjugated secondary antibody and developed with NBT-BCIP. (B) Secreted proteins detected by silver staining. Culture supernatant proteins of *Y. pestis*; wild type (WT) (vector; lanes 1and 2), *Y. pestis *ΔLcrG3 containing plasmids pBAD18 (vector; lanes 3 and 4), pAraG18 (LcrG, lanes 5 and 6), pJM174 (GAL4AD-LcrG; lanes 7 and 8), pLR1 (LcrG-GAL4AD, lanes 9 and 10), pLR2 (LcrGd2-6, lanes 11 and 12), pLR3 (LcrGpS; lanes 13 and 14), pLR4 (LcrGpI, lanes 15 and 16, pLR5 (LcrGpSI, lanes 17 and 18) were separated by SDS-PAGE in a 12.5% polyacrylamide gel and detected by silver staining.

**Figure 2:**
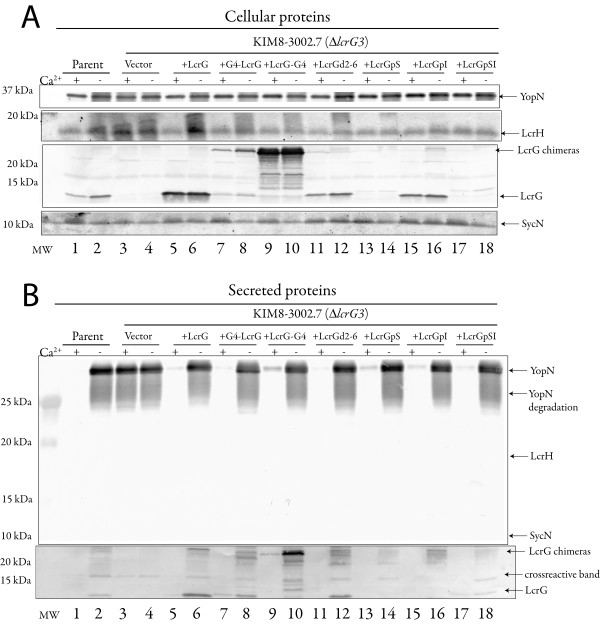
**LcrG secretion and expression by *Y. pestis *Δ*lcrG3 *with LcrG chimeras and N-terminal LcrG mutants**. Whole cell and cell-free culture supernatants were separated by SDS-PAGE in a 4–20% or 10.5–14% gradient polyacrylamide gels. Proteins were analyzed by probing with α-LcrG (4–20% gradient polyacrylamide gel) and α-YopN, α-LcrH, α-SycN (10.5–14% gradient polyacrylamide gel). Proteins were visualized by probing with alkaline phosphatase conjugated secondary antibodies and developed with NBT-BCIP. (A) Whole cell fractions, Immunoblots: Lanes 1 and 2 *Y. pestis *(wild type, WT), Lanes 3 and 4 *Y. pestis *Δ*lcrG3 *pBAD18, Lanes 5 and 6 *Y. pestis *Δ*lcrG3 *pAraG18, Lanes 7 and 8, *Y. pestis *Δ*lcrG3 *GAL4AD-LcrG, Lanes 9 and 10 *Y. pestis *Δ*lcrG3 *LcrG-GAL4AD, Lanes 11 and 12 *Y. pestis *Δ*lcrG3 *LcrGd2-6, Lanes 13 and 14 *Y. pestis *Δ*lcrG3 *LcrGpS, Lanes 15 and 16 *Y. pestis *Δ*lcrG3 *LcrGpI, Lanes 17 and 18 *Y. pestis *Δ*lcrG3 *LcrGpSI. Identical immunoblots were prepared and probed separately with α-YopN, α-LcrH, α-LcrG or α-SycN, the blots were scanned and strips used to present the data to conserve space. (B) Culture supernatants: Lanes 1 and 2 *Y. pestis *(wild type, WT), Lanes 3 and 4 *Y. pestis *Δ*lcrG3 *pBAD18, Lanes 5 and 6 *Y. pestis *Δ*lcrG3 *pAraG18, Lanes 7 and 8, *Y. pestis *Δ*lcrG3 *GAL4AD-LcrG, Lanes 9 and 10 *Y. pestis *Δ*lcrG3 *LcrG-GAL4AD, Lanes 11 and 12 *Y. pestis *Δ*lcrG3 *LcrGd2-6, Lanes 13 and 14 *Y. pestis *Δ*lcrG3 *LcrGpS, Lanes 15 and 16 *Y. pestis *Δ*lcrG3 *LcrGpI, Lanes 17 and 18 *Y. pestis *Δ*lcrG3 *LcrGpSI. Identical immunoblots were prepared, one was probed with α-LcrG and the second was sequentially probed and developed with α-SycN, then α-LcrH, and finally with α-YopN.

LcrG was shown to be secreted into the growth medium in the absence of calcium when *lcrG *was initially characterized in *Y. pestis *[2]. Interestingly, secretion of LcrG by *Y. enterocolitica *is not detected [17]. Secretion of LcrG by *Y. pseudotuberculosis *is not examined in the literature, though secretion of LcrG by *Y. pseudotuberculosis *IP2666 was not detectable (data not shown). Reasons for LcrG secretion in *Y. pestis *and not the enteropathogenic yersinae are unknown and any suggestions at this point would be purely speculative. The secretion of LcrG by *Y. pestis *could possibly relate to the strength of LcrG interaction with LcrV. However, Lawton et al reported a very strong interaction between LcrG and LcrV in Y.*pseudotuberculosis *when they characterized residues in LcrV involved in LcrG interaction [5] and the LcrG-LcrV interaction has not been studied in that detail in *Y. pestis *or *Y. enterocolitica*. Changes in the sequence of LcrG are not likely to account for the difference in secretion as the *Y. pestis *and *Y. pseudotuberculosis *sequences are identical and the *Y. enterocolitica *sequences are 96–98% identical, therefore any differences in LcrG-LcrV interaction would likely be LcrV dependent. LcrG has been shown to be primarily a cytoplasmic protein in *Y. pestis *[4]. However, the exact site of LcrG function remains to be elucidated. In this study, the ability of LcrG to function when LcrG secretion was blocked was examined to test the hypothesis that LcrG secretion may not be required for LcrG function. In order to determine the effect of LcrG secretion on LcrG function, LcrG chimeras to the GAL4 AD domain and mutants affected in LcrG secretion were constructed and analyzed for the ability to regulate Yops secretion, we reasoned that the addition of GAL4 AD domain should block LcrG secretion. An LcrG chimera with GAL4AD fused to LcrG's N-terminus [(GAL4AD-LcrG) constructed for a previous study [18]] and a new LcrG chimera with GAL4AD fused to LcrG's C-terminus (LcrG-GAL4AD) were introduced into an *lcrG *deletion strain of *Y. pestis *(Δ*lcrG3*, [19]) and the ability of the chimeras to regulate Yops secretion and the secretion of GAL4AD-LcrG and LcrG-GAL4AD, themselves, was examined. Results demonstrated that both GAL4AD-LcrG and LcrG-GAL4AD could transcomplement a Δ*lcrG3 *strain of *Y. pestis *for growth (data not shown), Yops expression (Fig. 1A) and Yops secretion (Fig. 1B). Expression of GAL4AD-LcrG and LcrG-GAL4AD restored Ca^2+^-regulation of Yops as evidenced by the Ca^2+^-regulation of Yops B, D, E and LcrV (Fig. 1A) in the transcomplemeted Δ*lcrG3 *strains. The GAL4AD chimera also restored control of Yops secretion as seen in the silver stained gel of culture supernatants (Fig. 1B). Both of the chimeric LcrG proteins were expressed well (Fig. 2A), the GAL4-LcrG appeared to be degraded to yield free LcrG and the LcrG-GAL4AD had many degradation products (Fig. 2A), possibly including free LcrG. Additionally, the LcrG-GAL4AD fusion was readily secreted (Fig. 2B, lane 10) and the (Fig. 2A) GAL4AD-LcrG expressing strain appeared to secrete LcrG (Fig. 2B, lanes 7–8). The generation and secretion of free LcrG from the LcrG GAL4AD chimeras and the secretion of the LcrG-GAL4AD chimera limits the ability to draw conclusions from this set of experiments, since low levels of LcrG are reported to have observable function [6]. However, these results are suggestive that LcrG may be a type III secretion substrate and that LcrG secretion may not be necessary for LcrG function. In addition, the ability of the LcrG-GAL4AD to be secreted is reminiscent of studies with YopE fusions that allow secretion of fusion proteins fused to the C-terminus of YopE [20-22].

Because the results with the GAL4AD constructs were not satisfactory, a second method to disrupt LcrG secretion was sought to further examine the role of LcrG secretion on LcrG function. The N-terminus of the Yop effector proteins has been shown to have a signal for secretion in *Yersinia *[9-11,22,23] and the mRNA of Yops may also serve as a signal [15,24,25]. Since our LcrG constructs were being expressed on plasmid constructs separate from native upstream DNA, the ability of mRNA signals to influence LcrG secretion was not examined. Accordingly, published studies on the YopE N-terminal proteinaceous secretion signal [9-11] were used to guide our mutational manipulation of *lcrG *to eliminate LcrG secretion. Plasmids expressing various mutant LcrG proteins under control of the *araBADp *were constructed. The mutant LcrG proteins comprise; a deletion of aa 2–6 (LcrGd2-6), a replacement of amino acids 2–6 of LcrG with poly-serine (LcrGpS), poly-isoleucine (LcrGpI), or an amphipathic sequence consisting of alternating serine/isoleucine residues (LcrGpSI). The resulting mutant LcrG constructs expressing LcrGd2-6, LcrGpS, LcrGpI or LcrGpSI were transformed separately into a Δ*lcrG3 *strain of *Y. pestis *and analyzed for LcrG and Yops expression and secretion. *Y. pestis *Δ*lcrG3 *transcomplemented with LcrGd2-6, LcrGpS, LcrGpI or LcrGpSI expressing plasmids all changed from calcium blind growth to calcium dependent growth (data not shown). Importantly, the Δ*lcrG3 *strain of *Y. pestis *transcomplemented with LcrGd2-6, LcrGpS, LcrGpI or LcrGpSI had restored Ca^2+ ^control of Yops expression (Fig. 1A) and Yops secretion (Fig. 1B) demonstrating LcrG function by the mutant LcrGd2-6, LcrGpS, LcrGpI and LcrGpSI proteins. Whole cell lysates from Δ*lcrG3 Y. pestis *transcomplemented with LcrGd2-6, LcrGpS, LcrGpI or LcrGpSI were separated by SDS-PAGE and immunoblotted with LcrG specific antiserum (α-LcrG) to visualize LcrG expression by the transcomplemented Δ*lcrG3 Y. pestis *strains (Fig. 2A). Δ*lcrG3 Y. pestis *strains transcomplemented with mutant LcrGs (LcrGd2-6, and LcrGpI) expressed LcrG at or near wildtype levels (Fig. 2B) demonstrating stable expression of LcrGd2-6 and LcrGpI. LcrGpS and LcrGpSI were weakly expressed (Fig. 2A; LcrG is barely visible in lanes 13, 14, 17 and 18). Immunoblots probed with α-LcrG from culture supernatants of Δ*lcrG3 Y. pestis *grown in the presence or absence of Ca^2+ ^demonstrated that LcrGpI was not detected in the culture supernatants (Fig. 2B) (some higher molecular bands are apparent in lane 16 (Fig. 2B) these are cross-reactive bands from the LcrV antisera that was used as a secretion control along with the LcrG antisera) suggesting that LcrGpI was not secreted. LcrGd2-6 was detected in the culture supernatant (Fig. 2B) demonstrating that amino acids 2–6 for LcrG are not required for LcrG secretion. LcrGpS and LcrGpSI were too weakly expressed for their secretion to be determined (Fig 2). The LcrG secretion results with LcrGd2-6, LcrGpS, LcrGpI and LcrGpSI suggest that amino acids 2–6 are not required for LcrG secretion. However, the composition of acids 2–6 of LcrG did affect LcrG secretion. Taken together, results with the LcrG GAL4AD chimeras and the N-terminal LcrG mutants support the hypothesis that LcrG secretion is not necessary for LcrG function. The results with LcrGpI provide the strongest evidence that LcrG secretion is not required for LcrG function as LcrGpI is expressed above wildtype levels (Fig. 2A; compare lanes 15–16 with lanes 1–2) and LcrGpI was not secreted unlike the case of the Δ*lcrG3 *strain transcomplemented with LcrG (Fig. 2B) where LcrG is well expressed and easily detected in culture supernatants. In this manuscript, LcrG secretion by wildtype *Y. pestis *was detectable. However, LcrG secretion has been variably observed [4] and the current results are consistent with previous studies on LcrG function in *Y. pestis *[2,4,6,18,26]. However, this variable LcrG secretion does questions whether LcrG is specifically secreted. To better define if LcrG is specifically secreted the presence of two known cytosolic proteins were examined. Whole cell fractions and culture supernatants were probed with antisera specific for the cytosolic chaperones LcrH and SycN (Fig. 2). Neither chaperone was secreted (Fig. 2B) consistent with the known behavior of T3SS chaperones. This result confirms that the presence of LcrG in culture supernatants is likely due to the action of the *Yersinia *T3SS and is not due to cell lysis during culture. Additionally the ability of the LcrG-GAL4AD chimera to be secreted and the disruption of LcrG secretion by mutation also suggest that LcrG is a T3SS substrate.

**Figure 3:**
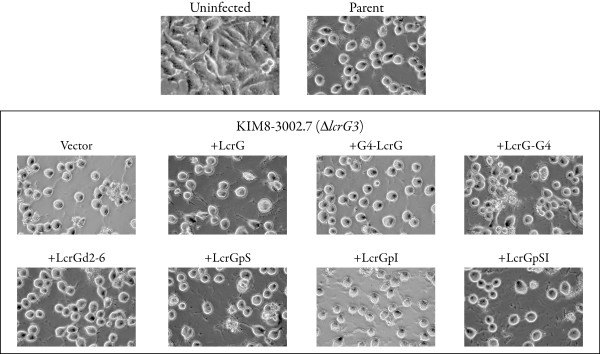
**HeLa cell infections by *Y. pestis *Δ*lcrG3 *with LcrG chimeras and N-terminal LcrG mutants**. HeLa cells were infected at an MOI of 30. Images were captured 3 hours post infection on an Olympus IX50 inverted microscope fitted with a Nikon D70 digital camera (magnification 400×) to document cell cytotoxcity.

**Figure 4:**
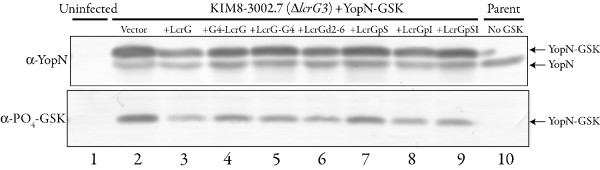
**Expression and translocation of YopN-GSK**. Translocation and phosphorylation of YopN-GSK in infected HeLa cells. Infected HeLa cell monolayers were solubilized with SDS-PAGE sample buffer and analyzed by SDS-PAGE in a 12.5% polyacrylamide gel and immunoblotted. Immunoblots were probed with phospho-GSK-3β antibodies that detect the GSK tag only when phosphorylated and α-YopN antibodies. Proteins were visualized by probing with alkaline phosphatase conjugated secondary antibodies and developed with NBT-BCIP.

The LcrG-GAL4 chimeras and the LcrG mutant proteins were also tested for the ability to support translocation of Yops into HeLa cells. Wildtype *Y. pestis*, Δ*lcrG3 Y. pestis *and Δ*lcrG3 Y. pestis *transcomplemented with GAL4AD-LcrG, LcrG-Gal4AD, LcrGd2-6, LcrGpS, LcrGpI or LcrGpSI all were tested for Yops translocation by examining HeLa cells infected with the various constructs. Infection of HeLa cells with yersiniae and examination of the cultured cells for cell 'rounding' has proven to be a very reliable indicator of Yops translocation [26,27]. The Δ*lcrG3 Y. pestis *strain transcomplemented with the mutant LcrG proteins (GAL4AD-LcrG, LcrG-Gal4AD, LcrGd2-6, LcrGpS, LcrGpI and LcrGpSI) or with vector alone were normal for Yops translocation (Fig. 3) compared to the wildtype *Y. pestis *strain (Fig. 3) after 3 h of contact. The ability of the mutant and chimeric LcrG proteins (GAL4AD-LcrG, LcrG-Gal4AD, LcrGd2-6, LcrGpS, LcrGpI and LcrGpSI) to support LcrG function both in vitro and in tissue culture demonstrates that the mutant LcrG proteins are functional to support Yops secretion regulation and the known enhancement of Yops translocation by LcrG [19]. Importantly for this study, mutant and chimeric LcrG proteins are functional to support Yops secretion and translocation even though LcrG secretion by Δ*lcrG3 Y. pestis *strains transcomplemented with LcrGpI is not observable, suggesting that LcrG secretion is not required to support translocation. To confirm the results with HeLa cell cytotoxicity, the ability of the Δ*lcrG3 *strains transcomplemented with wildtype and mutant LcrG proteins (GAL4AD-LcrG, LcrG-Gal4AD, LcrGd2-6, LcrGpS, LcrGpI and LcrGpSI) to translocate YopN was analyzed using a YopN-GSK construct that is phosphorylated only after translocation into the eukaryotic cell cytoplasm [28]. All of the mutant LcrG proteins supported translocation of YopN-GSK (Fig. 4) demonstrated by the appearance of PO_4_-YopN-GSK in the assay. Based on the intensities of the bands in Fig. 4, no significant differences in translocation efficiency are apparent.

The results presented in this study demonstrate that LcrG secretion is signaled at least in part by information in the N-terminus of LcrG as evidenced by the inability of the LcrGpI mutant to be secreted. This work is consistent with the observation of Lloyd et al. that poly-I can block secretion of T3SS substrates in the yersiniae [11]. However, since the LcrG Δ2–6 mutant could also be secreted, our results suggest that other information is retained in the LcrG Δ2–6 protein or in *lcrG *that guides LcrG into the T3SS. The results presented in this manuscript suggest that while LcrG is a substrate of the T3SS in *Y. pestis*, the ability of LcrG to be secreted by the T3SS is irrelevant to LcrG function.

## Conclusion

In this study, the relevance of LcrG secretion by the Ysc T3SS was examined. We found that LcrG could function after construction of LcrG and GAL4AD chimeric proteins, this was likely due to proteolytic release of LcrG from the chimera. However, those results prompted a deeper examination of LcrG secretion by the Ysc T3SS. In agreement with previous work [2] LcrG was found to be secreted by the Ysc T3SS in *Y. pestis*. Subsequent site directed mutagenesis of the putative T3S-signal in the N-terminus of LcrG resulted in LcrG mutants that were functional in Yops-secretion regulation and one stable LcrG mutant that not was secreted. The discovery of an LcrG mutant that was functional but not secreted suggests LcrG secretion while mediated by the Ysc T3SS is not necessary for LcrG function.

## Methods

### Bacterial strains and plasmids

The bacterial strains used in this study are listed in Table 1. *Y. pestis *was grown in heart infusion broth (HIB, Difco, Detroit MI) or on tryptose blood agar base (TBA, Difco) plates at 26°C for genetic manipulations. *Escherichia coli *strains were grown in Luria-Bertani broth (Difco) or on TBA plates at 37°C. When appropriate, bacteria were grown in the presence of carbenicillin at a concentration of 50 μg/ml.

**Table 1:** Strains and plasmids used in this study

**Strain**	**Relevant properties**	**Source or reference**
*E. coli *Novablue *Y. pestis*	*recA1 endA1 hsdR17*(r_K _m_K_^+^) *suE44 thi-1 gyrA96 relA1 lac *[F'*proA*^+ ^*B*^+ ^*lacI*^*q*^*ZΔM15*::Tn*10*]	Novagen
KIM8-3002	pCD1 (Lcr^+^) pMT1 Pla^- ^Sm^r^	[26]
KIM8-3002.7	KIM8-3002 Δ*lcrG3 *[LcrGΔ6-86]^a^	[19]

**Plasmids**		
pJM174	GAL4(768–881) AD LEU2 LcrG Ap^r^	[18]
pBAD18	*araBAD*p cloning vector, Ap^r^	[38]
pLR001	LcrG-GAL4(768–881) AD Ap^r^	This study
pLR002	pBAD18+*lcrG *[LcrGΔ2–6]^a ^(M Δ2–6 D E)	This study
pLR003	pBAD18+*lcrG *(polyserine aa 2–6)^b ^(M S S S S S D E)	This study
pLR004	pBAD18+*lcrG *(polyisoleucine aa 2–6)^b ^(M I I I I I D E)	This study
pLR005	pBAD18+*lcrG *(polyisoleucine/serine aa 2–6) b (M S I S I S D E)	This study
pAraG18	pBAD18+*lcrG *(wild type) (M K S S H F D E)	[26]

^a^Amino acids deleted are indicated. ^b^Amino acids changed are indicated.

### DNA techniques and plasmid constructions

Cloning methods were performed as described previously [29]. PCR fragments were purified using the QiaQuick PCR purification kit (Qiagen, Valencia, CA). Transformation of DNA into *E. coli *was accomplished by using commercially obtained competent cells (Novablue, Novagen, Madison, Wis.). Electroporation of DNA into *Y. pestis *cells was done as described previously [4]. Gene amplification was performed with Deep Vent (New England Biolabs, Beverly, Mass.) or *Taq *DNA polymerase (Eppendorf Scientific, Westbury, N.Y.). Plasmids used in this study are described in Table 1. Chimeric LcrG proteins were created by fusing the GAL4 activation domain (GAL4AD) to the N- or C-terminus of LcrG [6]. pLR01 (LcrG-Gal4) was constructed by amplifying LcrG with primers AraG-start (5' GGA ATT CAG GAG GAA AGG TCT TCC CAT TTG GAT 3') and AraG-back (5' CGC GGA TCC AAT ATT TTG CAT CAT CG 3'). The amplified sequences were digested with *Eco*RI and ligated into *Eco*RI- and *Sma*I-cleaved pBAD18. Gal4AD was amplified with primers described by Matson and Nilles [6]. Plasmids pLR02, pLR03, pLR04 and pLR05 were constructed by performing site-directed mutagenesis on pAraG18 [26]. Substitution mutations or deletion of amino acids (aa) 2–6 in the N-terminus of LcrG were constructed as indicated in Table 1. Site-directed mutagenesis was performed with *Pfu Turbo *DNA polymerase (Stratagene, La Jolla, Calif.) using the QuickChange Site-directed Mutagenesis Kit (Stratagene) according to the manufacturer's instructions. Oligonucleotide primers were synthesized by MWG Biotech (High Point, N.C.). Complementary oligonucleotides were designed to contain the desired mutation, flanked by unmodified sequence to anneal to the same sequence on opposite strands of the template plasmid. All mutations were confirmed by sequencing.

### Media and growth conditions

Plasmids expressing LcrG, LcrG with substitution mutations/deletions of aa 2–6, or the LcrG-GAL4 chimeras were introduced into the Δ*lcrG*3 mutant strain KIM8.3002-7 [19] and cultures were grown in TMH (a chemically defined medium) [30] with or without calcium. The cultures were shifted to 37°C and arabinose (0.2% w/v) was added at the same time to induce expression of LcrG from the vectors. After 4 hours of growth at 37°C, samples from the cultures were harvested and separated into whole-cell and cell-free culture supernatants as described previously [4]. Both fractions were analyzed by immunoblotting to assess protein expression and by immunoblotting or silver staining (Silver Snap II, Pierce, Rockford, IL) to assess protein secretion.

### Protein electrophoresis and immunodetection

Fractions corresponding to 0.05 A_620_·ml of bacterial whole cell or culture supernatants prepared in 2 × SDS-PAGE sample buffer were loaded for all protein samples. Proteins were separated by SDS-polyacrylamide gel electrophoresis (SDS-PAGE) according to the method described by Laemmli [31]. Proteins resolved by SDS-PAGE were silver stained or transferred to Immobilon-P membranes (Millipore Corp., Bedford, Mass.) using carbonate transfer buffer (pH 9.9) [2] for immunoblotting. Specific proteins were visualized using rabbit polyclonal antibodies specific for LcrG (α-LcrG [4]), YopB (α-YopB), YopD (α-YopD [32]), YopE (α-YopE), LcrV (α-LcrV [4]), YopN (α-YopN [33]), SycN (α-SycN [34]) and LcrH (α-LcrH [35]) as primary antibodies and alkaline-phosphatase conjugated secondary antibodies followed by development with 5-bromo-4-chloro-3-indolylphosphate/nitroblue tetrazolium NBT-BCIP [36]. Conditions used for immunodetection were the same for whole cell and supernatant fractions.

### HeLa cell infections

Yop translocation was monitored visually by cytotoxicity (cells rounding up) with cultured HeLa cells grown in Dulbecco's Modified Eagle Minimum essential medium (D-MEM) supplemented with 10% fetal calf serum (FCS, Invitrogen, Carlsbad, CA), penicillin, pyruvate and glutamine and grown at 37°C in a 5% CO_2 _atmosphere. HeLa cells were seeded into 24-well tissue culture plates (10^5 ^cells/well) and after the HeLa cells reached near confluency the growth medium was removed and the cells washed twice with L15 medium and placed in L15 for infection [26]. Next 30 μl of 10^5 ^CFU/μL of bacteria were added (MOI:30). The plates were centrifuged for 5 minutes at 25°C (300 × *g*) to allow cell contact. The plates were incubated at 37°C for 2–6 hours to check for cytotoxicity and photographed at 3 h.

### GSK phosphorylation to monitor translocation

*Y. pestis *strains carrying a pBAD33 derivative expressing YopN-GSK [28] were pre-induced with L-arabinose (0.2% w/v) for 1 h prior to infection and 0.2% arabinose (w/v) was maintained during the infection. HeLa cell monolayers were infected with *Y. pestis *strains at a multiplicity of infection (MOI) of 30 for 3 h at 37°C in L15 medium as described previously [6]. After 3 h, culture supernatants were decanted and the infected HeLa cells were lysed by the addition of 100 μl of 2 × SDS-PAGE lysis buffer containing mammalian cell protease (P-8340) and phosphatase (P-2850) inhibitor cocktails (Sigma). Samples were boiled for 5 min and analyzed by SDS-PAGE and separate identical immunoblots were probed with a GSK-3β (not shown; no. 9332, Cell Signaling Technology), a phosphospecific GSK-3β (no. 9336, Cell Signaling Technology) or an α-YopN antibody preparation. Secondary antibody (alkaline phosphatase-conjugated anti-rabbit immunoglobulin G) was diluted in TTBS containing 5% nonfat milk and 0.05% Tween 20 and incubated with the blots for 2 h. Blots were washed three times for five minutes and developed with BCIP-NBT.

### Image acquisition and production

All immnunoblots were scanned on an Epson 4490 Perfection scanner at 4800 dpi using VueScan software (v. 8.4.40; Hamrick Software, [37]). Micrographs were captured on a Nikon D70 digital camera in NEF format. The scanned blots and micrographs were imported into Adobe Photoshop (CS3, Adobe Software, San Jose, CA) the images were converted to grayscale and the auto levels function was applied. Final figures were assembled in Adobe Illustrator (CS3) and images were downsampled to the final resolution upon export to the PNG file format.

## Authors' contributions

JSM performed the initial studies with GAL4AD-LcrG, LDR performed subsequent experiments and wrote the draft of the manuscript, DMO performed the final experiments in the manuscript. MLN conceived of the study, supervised the work and edited the manuscript. All authors read and approved the final manuscript.
